# Alpha-1 Antitrypsin Deficiency and Bronchial Asthma: Current Challenges

**DOI:** 10.3390/biom15060807

**Published:** 2025-06-03

**Authors:** José Luis Lopez-Campos, Belén Muñoz-Sánchez, Marta Ferrer-Galván, Esther Quintana-Gallego

**Affiliations:** 1 Unidad Médico-Quirúrgica de Enfermedades Respiratorias, Instituto de Biomedicina de Sevilla (IBiS), Hospital Universitario Virgen del Rocío, Universidad de Sevilla, 41013 Sevilla, Spain; belen.munoz.sanchez.sspa@juntadeandalucia.es (B.M.-S.); marta.ferrer.sspa@juntadeandalucia.es (M.F.-G.); esther.quintana@telefonica.net (E.Q.-G.); 2 Centro de Investigación Biomédica en Red de Enfermedades Respiratorias (CIBERES), Instituto de Salud Carlos III, 28029 Madrid, Spain

**Keywords:** asthma, alpha-1 antitrypsin deficiency, prevalence, genetics, rare diseases

## Abstract

Alpha-1 antitrypsin deficiency (AATD) is a rare genetic condition classically associated with pulmonary emphysema and liver disease. However, the potential link between AATD and other respiratory diseases, particularly bronchial asthma, remains poorly understood and highly debated. This narrative review explores the current evidence regarding the epidemiological, clinical, and pathophysiological relationship between AATD and asthma. Data from prevalence studies show marked variability in the frequency of AATD-associated alleles among asthma patients, ranging from 2.9% to 25.4%, suggesting either a true association or selection biases. Conversely, asthma prevalence among AATD patients also varies widely, from 1.4% to 44.6%, with higher frequencies observed in countries with long-standing national registries. However, methodological inconsistencies and a lack of standardized diagnostic criteria limit the interpretation of these findings. Current evidence is insufficient to support a direct causal role for AATD mutations in asthma development, and no clear impact of AATD on asthma severity or prognosis has been established. Furthermore, there is no conclusive evidence that augmentation therapy is beneficial in asthma patients carrying AATD mutations. Despite these uncertainties, screening for AATD in selected asthma populations—especially those with severe or atypical phenotypes—may be warranted, as recommended by major respiratory societies. Future research should focus on large, well-powered, prospective studies that evaluate the potential pathophysiological interactions between AATD and specific asthma endotypes, particularly T2-low asthma. These efforts may help clarify the relevance of AATD mutations in asthma pathogenesis and identify potential therapeutic targets.

## 1. Introduction

Alpha-1 antitrypsin (AAT) is a glycoprotein predominantly synthesized in the liver, whose principal biological function is the inhibition of neutrophil elastase. By neutralizing this proteolytic enzyme, AAT plays a critical role in protecting pulmonary tissue from inflammation-induced damage and in preserving lung architecture and function. In addition to its protease inhibitory activity, AAT possesses notable anti-inflammatory and immunomodulatory properties. It contributes to the regulation of neutrophil activity, modulates cytokine production, and supports immune homeostasis. Consequently, alpha-1 antitrypsin deficiency (AATD) can result in progressive pulmonary disorders, including early-onset emphysema. Furthermore, the accumulation of misfolded AAT in hepatocytes may lead to liver pathology, such as cirrhosis and, in some cases, hepatocellular carcinoma.

Despite significant advancements in the clinical understanding and management of alpha-1 antitrypsin deficiency (AATD) in recent decades, numerous clinical questions remain unresolved. One of the current challenges in AATD management lies in its association with comorbidities beyond pulmonary emphysema and liver disease. The systemic implications of AATD are rooted in two key aspects. First, although alpha-1 antitrypsin (AAT) is primarily synthesized in the liver, it is also produced—albeit to a lesser extent—in other organs. Second, AAT exerts pleiotropic and systemic effects, meaning its deficiency could potentially disrupt the physiology of multiple organ systems. Accordingly, multidisciplinary teams would be required for a global approach to these patients if this systemic effect would be of clinical relevance [1].

Previous studies and case reports have explored the relationship between AATD and systemic clinical manifestations, including classic associations such as panniculitis [2] and systemic vasculitis [3]. Additionally, there is ongoing debate regarding the potential links between AATD and other systemic conditions, particularly cardiovascular diseases and malignancies, which represent major areas of interest [4,5]. In recent years, emerging hypotheses have also suggested possible associations between AATD and immunodeficiencies, metabolic disorders, and hematologic abnormalities [6,7,8]. Consequently, the medical literature underscores that the systemic impact of AATD remains incompletely understood, and future research may establish the relevance of AATD-related mutations in other comorbidities.

A particularly compelling aspect of AATD’s association with comorbidities is its relationship with respiratory diseases [9]. This potential connection presents a clinically significant challenge, both due to the overlapping symptoms between AATD and other pulmonary conditions and the long-term symptomatic and prognostic implications of their interaction. Among the most extensively studied respiratory diseases in this context are bronchiectasis, cystic fibrosis, bronchial asthma, sleep apnea, and pneumothorax [10,11,12,13]. While the established link between bronchiectasis and AATD has led to recommendations for AATD screening as part of the etiological workup for bronchiectasis [14,15], the role of AATD in other respiratory diseases remains controversial.

Bronchial asthma stands out as a respiratory disease of particular interest due to its high population prevalence, its occurrence across all age groups, and its potential for unfavorable progression if triggering and prognostic factors are not adequately controlled [16]. However, the association between AATD and asthma remains contentious [17]. This narrative review aims to evaluate the current state of research on the relationship between asthma and AATD, with the goal of informing clinical decision-making and encouraging further investigation in this field.

## 2. Prevalence of AATD in Patients with Asthma

The frequency of AATD-related alleles in patients diagnosed with bronchial asthma is summarized in Table 1. This table organizes the data by geographic region and date, given that the prevalence of AATD mutations is associated with geographic location, and asthma diagnostic criteria have evolved over time. The first formal study examining the frequency of AATD alleles in asthma patients was conducted in Norway in a group of 591 patients admitted due to an exacerbation of different respiratory diseases of which 39 were categorized as asthma [18]. This was a small initial study, biased by the fact that it involved exacerbated patients in whom AAT levels would be expected to increase due to the acute event. However, the high number of AATD mutations found (20.5%) signaled the need for further investigation.

The first study in stable asthma patients was conducted in Chicago, where 46 children with clinically diagnosed bronchial asthma were evaluated by measuring serum AAT levels via electro-immunodiffusion and protein phenotyping via electrophoresis. The authors found a 21.7% prevalence of carriers for at least one AATD allele, all of whom were heterozygous for the PI*S, PI*Z, PI*V, and PI*I alleles. Since then, studies assessing the frequency of AATD-related alleles in asthma patients have steadily increased (Table 1). Interestingly, it was not until more recently that specific mutations were described in patients with bronchial asthma [19,20]. Unfortunately, methodological inconsistencies across studies have made it difficult to obtain reliable allele frequency estimates in some cases [21,22,23,24].

Based on available data from published studies with clear prevalence rates of AATD alleles in asthma patients (Table 1), the frequency of these alleles varies considerably, ranging from 2.9% in the study by Aiello et al. [25] in Parma, Italy, to as high as 25.4% in the study by Colp et al. in Puerto Rico [26]. In the study by Aiello et al. [25], the authors examined 735 subjects with mild to moderate asthma and identified only 22 carriers of AATD mutations, with allelic combinations including PI*MS, PI*MZ, PI*MM_malton_, and PI*SS. In contrast, Colp et al. evaluated 105 cases with asthma exacerbations and found 14 carriers, all heterozygous for the PI*S, PI*Z, and PI*V alleles. Between these two studies, the remaining studies (Table 1) show considerable variability in results. Most studies have been conducted in Europe and the United States. Interestingly, the prevalence of AATD alleles in asthma patients in Italy is significantly lower than that observed in Spain. This finding is consistent with Spain’s strategic position in AATD allele distribution, characterized by a high frequency of the PI*Z allele—traditionally linked to Viking invasions in the Iberian Peninsula—as well as an elevated prevalence of the PI*S allele, which is believed to have originated near the Iberian Peninsula [27]. The United States exhibits an intermediate allele frequency, likely influenced by significant migratory flows following the discovery of the Americas by Spain in 1492. This migration may also explain the notably high prevalence observed in Puerto Rico as a result of Spanish immigration.

These findings are noteworthy because we know that the prevalence of PI*S and PI*Z alleles in the general population have a lower frequency [28,29]. In particular PI*S ranges from 7.3 to 31.3 per 1000 inhabitants in Northern Europe and from 18.6 to 185.1 per 1000 inhabitants in Western, Southern, and Central Europe. Similarly, PI*Z ranges from 0.0 to 45.1 per 1000 inhabitants in Northern Europe and from 7.3 to 29.7 per 1000 inhabitants in Western, Southern, and Central Europe [30]. These figures are lower than those reported in these AATD prevalence studies among asthma patients, suggesting either a potential association between AATD and asthma or a selection bias in these observational studies due to the inclusion of particularly vulnerable asthma populations.

**Table 1:** Studies evaluating the prevalence of AATD in subjects with asthma organized by country and date.

Study	Size	Origin of Data	Asthma Diagnosis	Diagnosis of AATD	Prevalence
Northern Europe					
Fagerhol MK, Hauge HE. Acta Allergol 1969 [18]	39	Norway	Physician diagnosed. Exacerbated	AAT phenotyping	8 cases (20.5%)
von Ehrenstein OS et al. Arch Dis Child 2004 [21]	2747	Germany ISAAC study [31]	Physician diagnosed or suggestive symptoms in children 9–11 yr	AAT plasma levels by nephelometry and PI*S and PI*Z genotypes by PCR	Not specified
van Veen IH et al. Respir Med 2006 [32]	122	The Netherlands	Severe asthma	AAT phenotypes by isoelectrofocusing	6 cases (4.9%)
Southern Europe					
Miravitlles M et al. Respir Med 2002 [33]	111	Spain	Symptomatic in the previous year >14 yr.	All cases with both: Hematic AAT levels by nephelometry AAT phenotypes by isoelectrofocusing	22 cases (19.8%)
Suarez-Lorenzo I et al. Clin Transl Allergy. 2018 [34]	648	Spain	Allergic asthma	AAT serum levels by nephelometry AAT genotypes by PCR	145 cases (22.3%)
Suarez-Lorenzo I et al. J Asthma 2022 [22]	648	Spain	Allergic asthma	AAT genotypes by PCR for the PI*Mmalton mutation	145 cases (22.3%)
Hernández-Pérez JM et al. Pulmonology. 2023 [35]	485	Spain	Physician diagnosed	AAT serum levels by nephelometry AAT genotypes by PCR + sequencing if discrepancy	117 cases (24.2%)
Aiello M et al. Respiration 2021 [36]	600	Italy	Mild to moderate asthma	AAT serum levels by nephelometry and AAT phenotypes by isoelectrofocusing if AAT < 113 mgr/dL or symptom/family history of AATD + sequencing if discrepancy	22 cases (3.6%)
Vianello A et al. J Allergy Clin Immunol Pract 2021 [37]	143	Italy	Severe asthma with biologics	AAT level < 1.1 g/L followed by phenotype by isoelectrofocusing or genotype by PCR.	10 cases (6.9%)
Aiello M et al. J Asthma 2022 [25]	735	Italy	Mild to moderate asthma	AAT serum levels by nephelometry and AAT phenotypes by isoelectrofocusing if AAT < 113 mgr/dL or symptom/family history of AATD + sequencing if discrepancy	22 cases (2.9%)
Northern America					
Hyde JS et al. Ann Allergy 1979 [38]	46	USA	Physician diagnosed	Serum AAT by electroimmunodiffusion and phenotypes	10 cases (21.7%)
Eden E et al. J Asthma 2007 [39]	285	USA LODO trial [40]	Physician diagnosed, >15 yr. Uncontrolled	Hematic AAT levels AAT phenotypes by isoelectrofocusing	35 cases (12.2%)
Ortega VE et al. J Allergy Clin Immunol Pract. 2025 [23]	1293	USA NHLBI SARP	Asthma symptoms confirmed by either: Methacholine test Bronchodilator reversibility	Gene sequencing	Not disclosed
Townley RG et al. Chest 1990 [41]	486	USA	Clinical presentation + spirometry reversibility	Serum AAT by radioimmunodiffusion + phenotype	70 cases (14.4%)
Other geographical areas					
Mousavi SA et al. Tanaffos 2013 [42]	43	Iran	Persistent asthma, >14 yr.	AAT serum levels by turbidimetry < 90 mg/dL	2 cases (4.6%)
Montealegre F et al. P R Health Sci J. 2006 [43]	105	Puerto Rico	Asthma exacerbation Physician diagnosed	Both: AAT serum levels by a fluorescence immunoassay AAT phenotypes by isoelectrofocusing	14 cases (13.3%)
Colp C et al. Arch Intern Med. 1990 [26]	55	Puerto Rico	Respiratory symptoms of asthma or COPD	AAT phenotypes	14 cases (25.4%)
Tural Onur S et al. Int J Chron Obstruct Pulmon Dis. 2023 [24]	209	Turkey	Physician diagnosed	AAT hematic levels by nephelometry + AAT genotype by Progenika on dried blood spots.	Not specified for asthma

## 3. Prevalence of Asthma in Patients with AATD

Equally interesting is an examination of studies assessing the frequency of bronchial asthma in patients with AATD. Table 2 provides a list of available studies on asthma prevalence in AATD patients, also organized by geographic region and publication date. In this case, the data are likely more compelling, as many of these studies derive from analyses of national or international AATD registries, which employ a specific and more consistent methodology, with well-defined patient selection criteria—typically including only severe AATD cases. Consequently, the analysis of mild AATD genotypes or heterozygous carriers tends to be underrepresented in many of these studies. Nevertheless, the frequency of asthma in AATD patients is also highly variable, ranging from 1.4% in New Zealand [44] to 44.6% in the Alpha1 Foundation registry in the United States [45]. The next lowest reported frequency of bronchial asthma comes from the study by Larsson et al., conducted in Sweden in 1978, which found an asthma prevalence of 3.2% among 246 AATD subjects [46]. This prevalence is relatively low compared to later studies conducted in the same country, particularly considering that the PI*Z mutation originated in Northern Europe, likely in Scandinavia [27]. The most straightforward explanation may relate to the diagnostic criteria for bronchial asthma, which, according to the authors, were based on the 1961 World Health Organization guidelines. Among the remaining European studies, the prevalence of AATD alleles in asthma patients ranges from approximately 10.8% in the United Kingdom [47] to 17.8% in Spain [48]. The only outlier is Italy, where the prevalence of asthma in AATD patients is lower than in the rest of Europe.

Interestingly, the highest prevalence rates of asthma in AATD patients are found in the United States (Table 2). In this country, data collection on AATD has been well-structured since the establishment of the National Heart, Lung, and Blood Institute registry in 1991, which was later continued by the Alpha1 Foundation registry starting in 1997 and remains active to this day. This represents over 30 years of continuous AATD patient registration. Although the prevalence of AATD in the U.S. is expected to be lower than in Europe, the consistency and methodology of this registry provide substantially higher data regarding asthma prevalence in the AATD population. Of note, a similar active registry in Europe—the EARCO (European Alpha-1 Research Collaboration) initiative, established in 2020—reports asthma frequencies consistent with other European studies, reinforcing an expected asthma prevalence of around 15% in AATD patients [63,64,65].

Interestingly, the reported asthma rates in AATD patients align closely with general population estimates—moving from less than five to over 20% worldwide [66]. These findings suggest that, overall, AATD patients exhibit asthma prevalence comparable to the general population, except in long-standing registries where asthma rates exceed population baselines. This discrepancy suggests a potential underrepresentation of asthmatic patients in European AATD registries.

## 4. AATD as a Risk Factor for Developing Asthma

Beyond the prevalence figures in the two clinical contexts mentioned above, there remain two open debates that must be resolved to assess the potential relationship between asthma and AATD. The first is whether AATD-associated mutations are a risk factor for developing bronchial asthma. The available evidence is truly limited, as an appropriate study design would require either a cohort study or a case–control study specifically aimed at evaluating the impact of AATD-associated mutations on the onset of asthma and therefore including in the study a population of asthmatic and non-asthmatic subjects in order to study the differences. As far as we are aware of, the only study addressing this issue stems from the ISAAC phase II study on the prevalence of bronchial asthma [21]. This study evaluated a randomized sample of 2747 children (48.8% of eligible subjects with available blood samples) aged 9–11 years. Although the prevalence of asthma did not differ among the identified AATD genotypes, the authors found a higher prevalence of bronchial hyperresponsiveness in PI*MZ subjects compared to other deficient genotypes, as well as increased bronchial hyperresponsiveness in children with AAT levels below 116 mg/dL. However, the number of cases was insufficient to detect statistically significant differences. Therefore, current evidence cannot confirm that AATD is a direct risk factor for the development of bronchial asthma. However, it is equally true that both asthma and AATD can coexist or be confused with each other due to symptomatic and functional overlap. Additionally, although AATD is primarily associated with Chronic Obstructive Pulmonary Disease, some patients may have asthma in addition to Chronic Obstructive Pulmonary Disease concurrently [67,68] and the relationship of AATD in this population has not been specifically studied.

## 5. Impact of AATD on Asthma

The second key issue regarding the relationship between AATD and asthma is whether carrying AATD mutations influences the clinical impact or prognosis of bronchial asthma. In this regard, several of the studies summarized in Table 1 also examine the symptomatic impact of AATD mutations on asthma on different clinical outcomes. However, these studies present significant methodological limitations that must be considered for a proper interpretation of their findings. First, some studies are outdated and rely on obsolete diagnostic criteria and asthma assessments, making it difficult to compare their results with current standards. Furthermore, the sample sizes in these studies are generally underpowered to detect significant differences in disease progression or prognostic parameters. In AATD research, power calculations suggest that between 300 and 500 patients with a minimum follow-up of 3 years would be required to demonstrate an effect on FEV1 decline [69], whereas mortality studies would need at least 342 subjects per group over 5 years in cases of severe AATD [70]. However, most available studies fail to meet these thresholds, limiting their ability to establish robust associations. Another relevant consideration is that the genotypes evaluated do not always correspond to the most severe AATD variants, which may lead to an underestimation of their true clinical impact. Additionally, only PI*S and PI*Z alleles are often the only alleles explored, excluding the impact of other less frequent severe mutations. Furthermore, many of these studies were not specifically designed to investigate the relationship between AATD and asthma; rather, they consist of post hoc analyses of prior research, introducing potential biases. From a statistical standpoint, the analyses performed are predominantly bivariate and exploratory, lacking the rigor required for multivariate hypothesis confirmation while accounting for confounding factors such as smoking history. Moreover, these studies have not systematically addressed the distinction between T2-high and T2-low asthma endotypes—a critical factor in disease heterogeneity. Finally, although these investigations describe baseline cohort characteristics, they fail to assess longitudinal disease progression, thereby precluding meaningful conclusions about the natural history of asthma in AATD patients. In light of these limitations, future research should employ prospective designs with sufficient statistical power and carefully characterized populations to accurately assess the contribution of AATD mutations to asthma clinical impact and prognosis.

Despite these limitations, several observed associations have emerged that may generate testable hypotheses for future study designs. One notable finding concerns the relationship with atopy. This potential association was first identified in two studies conducted by the same research group in Parma, Italy, which analyzed a large patient cohort. Their results demonstrated that the prevalence of atopy was significantly higher in patients with AATD compared to non-AATD controls [25,36]. However, this relationship has not been consistently shown by previous [38] nor subsequent studies [37] by other groups.

Another noteworthy aspect is the potential association between AATD mutations and the risk of exacerbations. While a previous study found no association between AATD mutations and asthma exacerbations in children [38], only one study to date has explored this clinical outcome in adults [23]. This recent investigation analyzed two cohorts of patients with asthma of varying phenotypes and severity. The authors observed that individuals with the P*MZ genotype and a history of smoking experienced a higher frequency of asthma-related emergency department visits, hospitalizations in the previous year, and lifetime admissions to intensive care units. Moreover, in the combined cohort of white patients irrespective of smoking status, MZ heterozygotes showed an increased frequency of asthma-related hospitalizations and ICU admissions over their lifetime. The authors propose an asthma-specific “protective threshold” for exacerbations—defined by serum AAT levels above 139 mg/dL—which is notably higher than the threshold typically used for emphysema treatment. However, this effect was identified by analyzing selected subgroups within the larger cohort, highlighting the need for further research to fully elucidate this potential relationship and to better understand the underlying factors influencing this association.

More disappointing is the potential association between AATD mutations and clinical outcomes related to pulmonary function. There is a clear scarcity of studies demonstrating a significant, clinically relevant, and consistent relationship between the presence of AATD mutations and lung function in patients with bronchial asthma—this holds true both in studies including patients across a range of disease severity [26,39], in those focusing specifically on more severe cases [42], and in pediatric populations [21]. One study, conducted by Aiello M et al., reported an association, observing a lower forced vital capacity and a higher residual volume to total lung capacity ratio in individuals with AATD [36]. In this context, AATD mutations have not been clearly linked to asthma severity [34], nor to the development of persistent airflow obstruction in the studies that have evaluated this outcome [32].

An intriguing aspect of functional assessment in patients with asthma is the evaluation of bronchial hyperresponsiveness. An early study conducted in children, though limited by sample size, reported that inhalation of isoproterenol led to a significantly greater improvement in airway obstruction in non-mutated asthmatic children compared to those carrying AATD mutations [38]. However, subsequent pediatric studies have found no significant increase in bronchial hyperresponsiveness in children with low AAT levels compared to those with normal levels [21]. This discrepancy highlights the need for further investigation to clarify the potential impact of AATD mutations on bronchial hyperresponsiveness.

The symptomatic burden of asthma and the use of asthma medications have also not shown a consistent association with the presence of AATD mutations [21,33,37,38]. Similarly, these mutations have not been clearly linked to asthma severity [33,34]. Finally, analytical parameters such as blood eosinophil counts, total serum IgE levels, and fractional exhaled nitric oxide concentrations have not been found to be significantly altered in asthmatic patients carrying AATD-related mutations [33,37,41].

In conclusion, the available studies examining the relationship between AATD mutations and bronchial asthma are methodologically limited and do not allow for definitive conclusions. Nevertheless, some intriguing hypotheses have emerged from these analyses, warranting further investigation through future studies with adequately powered sample sizes and robust multivariate designs to enable the generation of reliable and clinically meaningful conclusions.

## 6. Augmentation Therapy for AATD and Bronchial Asthma

To date, no clinical trial or observational study has been specifically designed to evaluate the effect of augmentation therapy on patients with AATD and bronchial asthma. Further, augmentation therapy trials do not specifically include patients with asthma [71,72,73]. Consequently, there are currently no recommendations regarding augmentation therapy for AATD in patients with AATD and asthma [74]. Nevertheless, some findings warrant further discussion. In an animal model study, one research group uncovered novel pathological mechanisms and identified potential new therapeutic targets for asthma. Specifically, they demonstrated that AAT may alleviate inflammation and oxidative stress by suppressing autophagy in the context of asthma, thereby potentially improving the disease [75].

Interestingly, in real-world clinical practice, only isolated case reports have been published. In 2008, Blanco et al. reported the case of an asthmatic woman with the PIMZ genotype in whom augmentation therapy halted the decline in FEV1, reduced the number of exacerbations, eliminated the need for oral corticosteroids, and improved quality of life [76]. Subsequently, in Padua, Italy, a case was described involving a 31-year-old woman with the PISZ genotype and severe persistent asthma refractory to treatment, who was also pregnant. The patient experienced frequent exacerbations, and following the initiation of augmentation therapy, there was an improvement in lung function parameters and a reduction in exacerbation frequency [77].

Beyond these two anecdotal cases, no clinical trial has specifically focused on investigating the effect of augmentative therapy on bronchial asthma. Only one study has included patients with asthma [78], but it enrolled only six such cases, thus not truly exploring this population. Therefore, on the basis that supportive data are sparse and anecdotal, our current practice is not to offer augmentation therapy for asthma.

## 7. Summary and Research Questions

**Figure 1:**
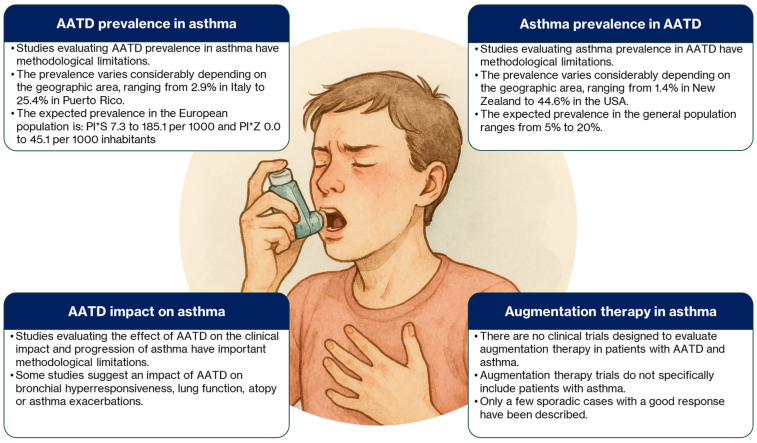
Summary of the main points in the association between asthma and AATD.

In conclusion, the analysis of the available evidence on the relationship between AATD and bronchial asthma raises more questions than it answers (Figure 1). Although some studies have explored a potential epidemiological link between these two clinical conditions, their actual clinical impact remains to be demonstrated. It is well known that AATD promotes a predominantly neutrophilic inflammatory profile, suggesting that a possible association with T2-low asthma could be a particularly relevant avenue for investigation. However, none of the existing studies have specifically addressed this pathogenic pathway in asthma or its connection to AATD. Moreover, the methodological limitations noted in the association studies preclude any definitive conclusions regarding a clinically meaningful link between the two conditions. That said, if a clear association existed, one might expect at least some signal to have emerged from the existing evidence. Therefore, important areas of uncertainty remain, warranting further investigation to elucidate the clinical impact of AATD on asthma. Such research should employ appropriately powered studies, adjusted for relevant covariates, to examine the association both in the general asthma population and across clinically relevant asthma subtypes.

Some of the key research questions that need to be addressed include: (1) a large-scale population-based evaluation of the epidemiological association between AATD and asthma; (2) a properly powered prospective study to assess the impact of AATD on the clinical course, progression, and prognosis of bronchial asthma; and (3) a clinical trial to evaluate the potential role of augmentation therapy on clinical outcomes in patients with bronchial asthma associated with AATD. These efforts must necessarily take into account the diverse clinical phenotypes of asthma and the wide range of AATD mutations in order to gain a comprehensive understanding of the potential relevance of this association.

Despite all the aforementioned considerations, the World Health Organization in 1997, the American Thoracic Society in 2003, and the European Respiratory Society in 2017 recommended screening for genetic AAT deficiency in patients with asthma [79,80,81]. Unfortunately, the current editions of major clinical guidelines on bronchial asthma—such as those issued by GINA and various national health authorities—do not include recommendations for screening or diagnosing alpha-1 antitrypsin deficiency (AATD) in patients with asthma, even in cases where bronchial obstruction is not fully reversible [16,82]. Given the goal of reducing the underdiagnosis of AATD [83], the simplicity of current screening methods, and the increasing accessibility of genetic testing, it may not be unreasonable to recommend evaluating the presence of AATD in asthma—particularly in severe asthma phenotypes. Nevertheless, the clinical implications of such findings still need to be thoroughly assessed before any firm clinical or therapeutic recommendations can be made regarding the management of AATD in patients with bronchial asthma.

## Figures and Tables

**Table 2:** Studies evaluating the prevalence of asthma in subjects with AATD organized by country and date.

Study	Size	Origin of Data	Type of AATD	Asthma Diagnosis	Prevalence
Northern Europe					
Fähndrich S et al. COPD 2015 [49]	1066	Germany AATD registry	Severe AATD: AAT < 50 mg/dL with PI*ZZ or other deficient allelic variants	Physician diagnosed	170 cases (15.9%)
Chorostowska-Wynimko J et al. COPD 2015 [50]	55	Poland AATD registry	Severe AATD PiZ phenotype	Physician diagnosed	9 cases (16.3%)
Larsson et al. Acta Med Scand 1978 [46]	246	Sweden	Severe AATD PiZ phenotype	According to the WHO 1961 definition	8 cases (3.2%)
Piitulainen E, Sveger T. Thorax 2002 [51]	98	Sweden	PiZ phenotype	Physician diagnosed	15 cases (15.3%)
Piitulainen E, Tanash HA. COPD 2015 [52]	1553	Sweden AATD registry	PiZZ, PiZNull, or PNullNull phenotypes	Physician diagnosed	176 cases (11.3%)
Tobin MJ et al. Br J Dis Chest 1983 [47]	129	United Kingdom	PiZ phenotype	Physician diagnosed	14 cases (10.8%)
Southern Europe					
Luisetti M et al. COPD 2015 [53]	422	Italy AATD registry	Pi*ZZ, Pi*SZ, and carriers of rare deficient variants.	Physician diagnosed	21 cases (4.9%)
Ferrarotti I et al. Pulmonology 2025 [54]	281	Italy AATD registry	Pi*ZZ, Pi*SZ, and carriers of rare deficient variants.	Physician diagnosed	15 cases (5.3%)
Lara B, Miravitlles M. COPD 2015 [48]	448	Spain AATD registry	Pi*ZZ, Pi*SZ, and carriers of rare deficient variants.	Physician diagnosed	80 cases (17.8%)
Lara B et al. Arch Bronconeumol 2017 [55]	511	Spain AATD registry	Pi*ZZ, Pi*SZ, and carriers of rare deficient variants.	Physician diagnosed	57 cases (11.1%)
Torres-Duran M et al. ERJ Open Res 2022 [56]	405	Spain EARCO registry [57]	Any mutation not carrying a PI*M allele	Physician diagnosed	55 cases (13.5%)
Northern America					
Eden E et al. AJRCCM 1997 [58]	43	USA NHLBI registry	AAT < 11 µM PI*ZZ or PI*ZNull	One of: History of attacks of wheezing associated with shortness of breath Spirometric evidence of a bronchodilator response Presence of atopy upon skin testing Total serum IgE above 100 IU/mL	11 cases (25.5%)
McElvaney NG et al. Chest 1997 [59]	1129	USA NHLBI registry	AAT < 11 µM PI*ZZ or PI*ZNull	Not specified	350 cases (31.0%)
Eden E et al. Chest 2003 [60]	1052	USA NHLBI registry	AAT < 11 µM PI*ZZ or PI*ZNull	All of: A ≥ 12% improvement in FEV1 of at least 200 mL after bronchodilator on any visit Had a history of more than one attack of wheezing with shortness of breath Reported a doctor’s diagnosis of asthma or allergy	220 cases (20.9%)
Eden E et al. Respir Med 2006 [45]	757	USA Alpha1 Foundation registry	Any mutation	Physician diagnosed	338 (44.6%)
DeMeo DL et al. Thorax 2007 [61]	378	USA	PI*ZZ	Physician diagnosed	140 cases (37.0%)
Kelbel T et al. J Allergy Clin Immunol Pract 2017 [62]	226	USA	Severe AATD: ZZ, SZ, ZNull, and FZ	Physician diagnosed	64 cases (29.6%)
Other geographical areas					
Miravitlles M et al. Respir Res 2022 [63]	1044	International EARCO registry [57]	Any mutation not carrying a PI*M allele	Physician diagnosed	158 cases (15.1%)
Miravitlles M et al. Eur Respir J 2023 [64]	629	International EARCO registry [57]	PI*ZZ	Physician diagnosed	88 cases (14.1%)
Martin T et al. Respir Res 2024 [65]	634	International EARCO registry [57]	PI*SS and PI*ZZ	Physician diagnosed	109 cases (17.1%)
Janus ED et al. Lancet 1985 [44]	69	New Zealand	PiZ phenotype	Not specified	1 case (1.4%)

## Data Availability

No new data were created or analyzed in this study.

## Abbreviations

The following abbreviations are used in this manuscript:

AATD	alpha-1 antitrypsin deficiency
AAT	alpha-1 antitrypsin
EARCO	European Alpha-1 Research Collaboration
